# Zebrafish: A Resourceful Vertebrate Model to Investigate Skeletal Disorders

**DOI:** 10.3389/fendo.2020.00489

**Published:** 2020-07-31

**Authors:** Francesca Tonelli, Jan Willem Bek, Roberta Besio, Adelbert De Clercq, Laura Leoni, Phil Salmon, Paul J. Coucke, Andy Willaert, Antonella Forlino

**Affiliations:** ^1^Biochemistry Unit, Department of Molecular Medicine, University of Pavia, Pavia, Italy; ^2^Department of Biomolecular Medicine, Center of Medical Genetics, Ghent University-University Hospital, Ghent, Belgium; ^3^Bruker microCT, Kontich, Belgium

**Keywords:** zebrafish, skeletal system, x-ray, microCT analyses, imaging techniques, skeletal diseases

## Abstract

Animal models are essential tools for addressing fundamental scientific questions about skeletal diseases and for the development of new therapeutic approaches. Traditionally, mice have been the most common model organism in biomedical research, but their use is hampered by several limitations including complex generation, demanding investigation of early developmental stages, regulatory restrictions on breeding, and high maintenance cost. The zebrafish has been used as an efficient alternative vertebrate model for the study of human skeletal diseases, thanks to its easy genetic manipulation, high fecundity, external fertilization, transparency of rapidly developing embryos, and low maintenance cost. Furthermore, zebrafish share similar skeletal cells and ossification types with mammals. In the last decades, the use of both forward and new reverse genetics techniques has resulted in the generation of many mutant lines carrying skeletal phenotypes associated with human diseases. In addition, transgenic lines expressing fluorescent proteins under bone cell- or pathway- specific promoters enable *in vivo* imaging of differentiation and signaling at the cellular level. Despite the small size of the zebrafish, many traditional techniques for skeletal phenotyping, such as x-ray and microCT imaging and histological approaches, can be applied using the appropriate equipment and custom protocols. The ability of adult zebrafish to remodel skeletal tissues can be exploited as a unique tool to investigate bone formation and repair. Finally, the permeability of embryos to chemicals dissolved in water, together with the availability of large numbers of small-sized animals makes zebrafish a perfect model for high-throughput bone anabolic drug screening. This review aims to discuss the techniques that make zebrafish a powerful model to investigate the molecular and physiological basis of skeletal disorders.

## Introduction

**Figure 1:**
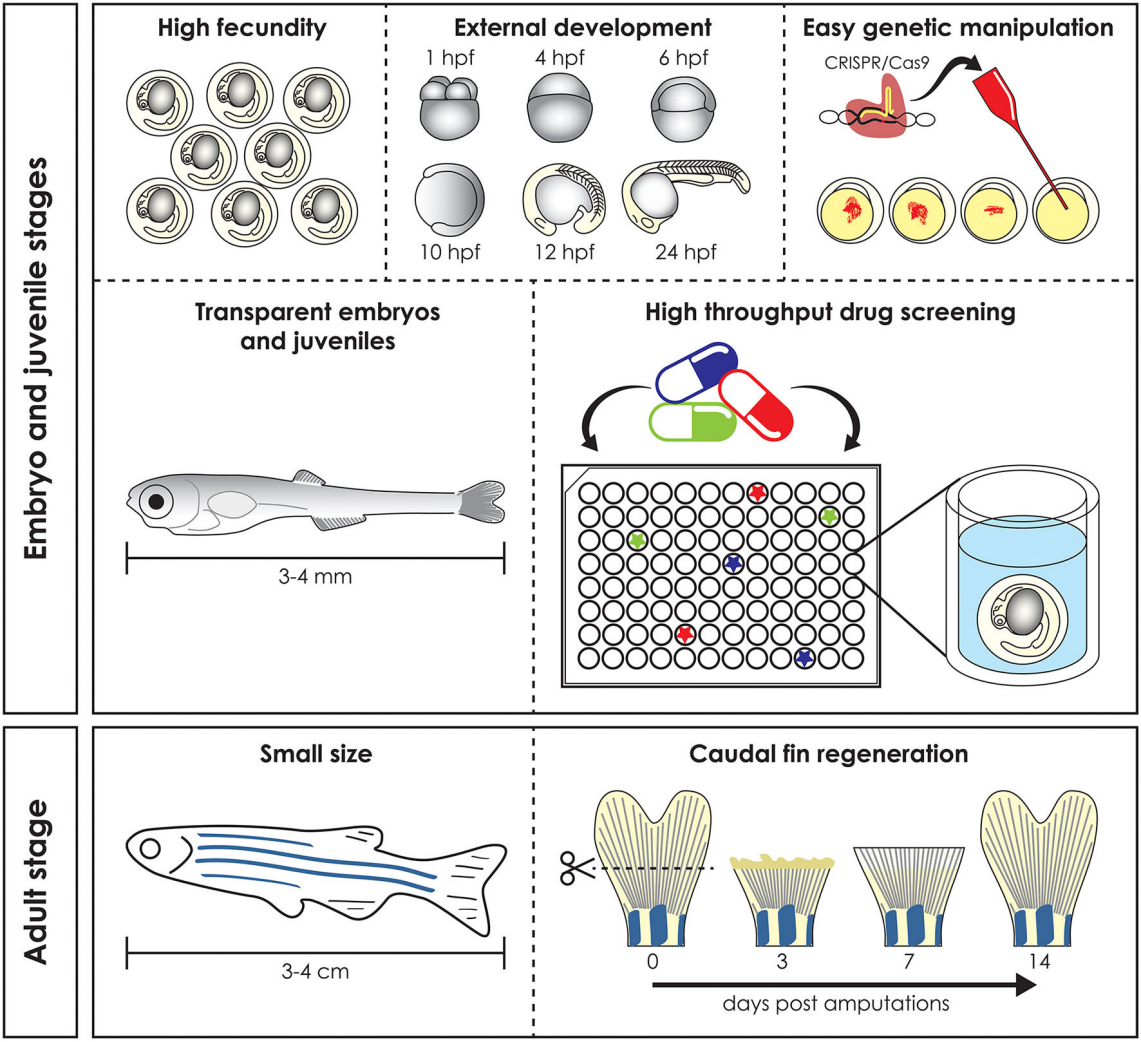
Advantages of the zebrafish model. Zebrafish has several advantages compared to mammal models. High fecundity and external fertilization and development allow easy genomic manipulation, transparent early life stages guarantee *in vivo* imaging and skin permeability makes them suitable for high throughput drug screening (top). Adult zebrafish reaches a maximum size of 3–4 cm and this make it easy and cheap to keep it in large numbers, reducing the husbandry cost (bottom left). Finally, zebrafish is used as a vertebrate model to study regeneration, due to its ability to regenerate different organs, such as the caudal fin, which is completely regenerated 14 days post amputation (bottom right). hpf, hours post fertilization.

Preclinical animal models can be used to elucidate gene and protein function in ways often impossible in humans, by means of genome sequencing, advances in DNA manipulation and high resolution live-imaging (1). Mammals such as mice and non-human primates are traditionally the preferred models for biomedical research due to their close evolutionary relationship with humans. However, their use is costly and studies at early developmental stages raise ethical concerns. Furthermore, in most countries the adoption of the “Three R's” principles: Replacement, Reduction, and Refinement (2) for animal research is mandatory and encourages the use of alternative models, such as *Danio rerio* (zebrafish), *Xenopus laevis/tropicalis* (clawed toad), *Drosophila melanogaster* (fruit fly), and *Caenorhabditis elegans* (nematode). In these organisms *in vivo* techniques can be applied with the simplicity and versatility of *in vitro* assays and therefore they are frequently used in fundamental and biomedical research to quickly define gene functions and to develop novel therapeutic options (3). Zebrafish, the most frequently employed non-mammalian vertebrate animal model, is a freshwater bony fish, belonging to the Cyprinidae family and to the Teleostei infraclass of ray-finned fish which arose ~340 million years ago (4). This species was initially described by the Scottish physician and naturalist Hamilton (5) in a survey on South Asian flora and fauna. Starting from the pioneering research of George Streisinger in the 70s−80s, who was the first to clone a zebrafish and in this way demonstrated the easy genetic manipulation of this species (6), zebrafish became a powerful model organism for developmental studies, genetic research, drug and toxicology screenings and for understanding tissue regeneration and repair (7–9). In contrast to other vertebrate models such as mice, fertilization occurs externally, which together with transparency and rapid embryo to larval transition permits easy access and visualization of development (10) (Figure 1). Moreover, due to its rapid growth, a recognizable and complete vertebrate body plan is already in place by 1 day post fertilization (dpf) and embryogenesis is complete by 3 dpf (11). In contrast to other vertebrate models such as rodents, the small size and large number of offspring of zebrafish allow for increased sample numbers, thereby increasing the statistical power of experiments (3). Finally, the relatively low husbandry cost further contributed to the increasing popularity of the zebrafish as a model for research (11).

Besides developmental studies, the zebrafish is an established research model in many other research fields. During the last 20 years, the zebrafish has proven itself as a useful model to study disease mechanisms (1). This is due to its physiological relevance and genetic tractability to model genetic variation in humans. Compared to mammalian model organisms, the zebrafish genome underwent an extra (third) whole duplication about 350 million years ago, with the result that for many genes in humans, there may be two copies (paralogues) in zebrafish. Despite this there is a relatively high level of genome conservation between zebrafish and humans with more than 70% of human protein-coding genes having at least one zebrafish ortholog. The haploid zebrafish genome has 25 chromosomes containing 1.7 billion base pairs (4). Various forward and reverse genetic approaches have been applied to generate mutant lines that mimic many different human diseases, including skeletal diseases ranging from secondary osteoporosis (OP) to rare disorders such as osteogenesis imperfecta (OI) (12–20). A major benefit of zebrafish is the simplicity of combining mutant and transgenic lines that express fluorescent reporter proteins under the control of responsive elements for signaling pathways or promoters of cell-type-specific markers. This in turn allows for *in vivo* investigation of the effect of a disease mutation on the spatio-temporal expression of specific genes, and on cell differentiation and signaling pathways.

Zebrafish larvae have been intensively used for pharmacological and toxicological screens, because of their small size (easy distribution in microtiter well plates), high abundance and their ability to absorb small compounds from the water through the skin and gills (21). Together with the availability of many different disease models, the zebrafish is a unique tool to develop novel targeted pharmacological approaches (Figure 1) (21).

Finally, their ability to regenerate some cells and tissues, such as fins and scales, makes the zebrafish a valuable model for understanding organ repair mechanisms during healthy and pathological conditions (Figure 1) (22).

This review, after providing a brief overview of zebrafish bone biology, will focus on the description and use of the various techniques and approaches which make *Danio rerio* a powerful model organism to investigate the molecular and physiological basis of skeletal disorders.

## Zebrafish Bone Biology

### The Skeleton

Skeletal development and gene expression and the general inventory of bone types are conserved between zebrafish and mammals, nevertheless few differences need to be considered when using this animal as model for skeletal study. Osteocytes are not present in all bones and/or at all developmental stages, endochondral ossification is rare in zebrafish and vertebral body do not build on a cartilaginous anlage (23, 24). The common perception of mammals being more complex than “lower” organisms, such as teleosts, is false, especially concerning the skeleton. Certain characteristics of the teleost skeleton are more advanced and elaborate compared to mammals, such as the zebrafish skull that contains at least twice the number of bones (24). At the tissue level, the mammalian skeleton mostly consists of cellular bone and hyaline cartilage. While other types of bone, such as hyperostotic and acellular bone and cartilage (i.e., fiber cartilage), can be present in mammalian skeletons, they are often associated with pathological processes. However, in teleosts many different bone and cartilage types with different cellularity and matrix composition exist in wild type conditions not related to disease (25). The zebrafish skeleton consists of a dermal skeleton and an endoskeleton. Scales, polarized structures of the exoskeleton, teeth, and fin rays are part of the dermal skeleton and are distinctive as skeletal structures in their ability to regenerate (25–27). In fish, teeth, scales, and fin rays can all be traced back in evolution to a single structure, called the odontode (28), and they arise at the epithelial-mesenchymal border (29, 30). It has been shown that the mesenchymal tissues that engender these skeletal elements have a neural crest origin (29, 31, 32).

The endoskeleton consists of cranial, axial, and appendicular skeletal elements (33). As in all vertebrates, the zebrafish cranial skeleton arises mostly from the cranial neural crest, while the appendicular skeleton develops from somite-derived paraxial mesoderm (31, 33). In contrast with tetrapods, in which vertebral centrum formation is controlled by somites patterned along the vertebral column, in teleosts the notochord has an instructive role in vertebral centrum patterning as the centra start out as mineralization foci in the notochord sheath (34, 35).

### Skeletal Cells

**Figure 2:**
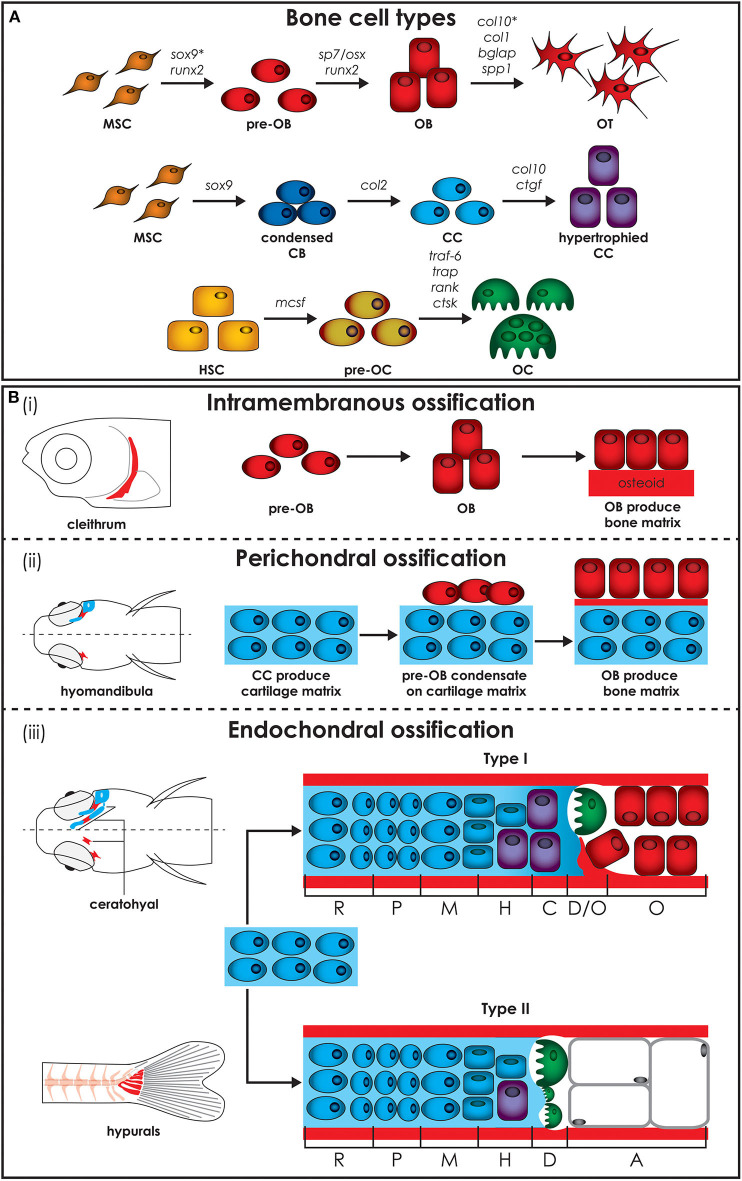
Zebrafish bone cells and ossification types. **(A)** Bone is formed by osteoblasts and osteocytes, while cartilage is formed by chondroblasts and chondrocytes, and both bone and cartilage are degraded by osteoclasts. All bone cell types develop from progenitors similar to the mammalian counterpart and share similar gene expression profiles (genes are indicated above arrows). Note however that HSCs in zebrafish are not present in the bone marrow but in the head kidney. In addition, the genes for collagen X, encoded by *col10*, and SRY-box transcription factor 9 (indicated by*), encoded by *sox9*, are expressed during osteoblasts differentiation in zebrafish, but not in humans. **(B)** Three types of ossification are present in zebrafish: (i) intramembranous ossification, (ii) perichondral ossification, present in teleosts but not in humans, and (iii) endochondral ossification. (i) During intramembranous ossification mesenchymal stem cells condensate and differentiate into pre-osteoblasts and finally into mature osteoblasts that deposit bone matrix (osteoid) that subsequently mineralizes. (ii) Perichondral ossification starts at the surface of a cartilaginous template where osteoblasts deposit bone matrix without replacing the cartilage. (iii) Endochondral ossification is the process by which growing cartilage is replaced by bone to allow the skeleton to grow. For ossification to start, matrix surrounding the chondrocytes must calcify so that osteoclasts can break down the cartilage. In teleost two types of endochondral ossification exist. Type I endochondral ossification, typical in the ceratohyal, resembles the mammalian endochondral ossification process. This is characterized by a hypertrophic zone, where the cartilage matrix calcifies, followed by a degradation zone where osteoclasts (also referred to as chondroclasts) degrade the cartilaginous matrix, and a bone formation zone. Type II ossification, in the hypurals, is characterized by a lack of the calcification and ossification zones, leading to tubular concave bones filled with adipose tissue. Schematics based on detail description in Weigele and Franz-Odendaal (36). A, adipose zone; C, calcification zone; CB, chondroblasts; CC, chondrocytes; D, degradation zone; H, hypertrophic zone; HSC, hematopoietic stem cell; M, maturation zone; MSC, mesenchymal stem cell; O, ossification zone; OB, osteoblasts; OC, osteoclasts; OT, osteocytes; P, proliferation zone; R, rest zone.

The teleost and mammalian skeletal systems share similar cell types (Figure 2A). In cartilage there are (i) chondroblasts as the cartilage forming cells and (ii) chondrocytes maintaining the cartilage matrix. In bone there are (i) osteoblasts as the bone forming cells, (ii) osteocytes that act as the mechanosensors regulating osteoblast and osteoclast activity and (iii) osteoclasts which are the bone resorbing cells (24, 37). Similar to mammals, teleost skeletal histogenesis involves the differentiation of chondroblasts and osteoblasts, that secrete the collagen extracellular matrix, from mesenchymal stem cells (38, 39). Both in mammals and fish, skeletal cells are formed by a complex interplay of intracellular molecular pathways and secreted factors that regulate the timing, location, and pathway by which bone cells differentiate (40–42). Although not investigated in mammals before, in zebrafish osteoblasts are present in clusters at the end of growing bones and can be classified in two different groups (type I and type II) based on cell cluster size, location, and nuclei shape, although they have overlapping functions (36). Type I osteoblasts are located at the edges of growing flat bones, such as the dentary, maxillary, and frontal bone, in large clusters with more than 25 cells with a wide oval, round, or irregularly shaped nucleus. Laterally to these cells there is a zone of differentiating osteoblasts where cells are smaller and more elongated, assuming the typical spindle shape of osteoblast-like cells, which cover all zebrafish bones with a monolayer at the level of the perichondrium (36). Type II osteoblast clusters are smaller (4–12 cells) and are distributed throughout the skeleton. These osteoblasts have a reduced size, elongated nucleus and are present throughout the bony trabecular network of spongy bones. Type II osteoblast clusters can also be detected at the edges of cartilage break down zones and lateral to the epiphysial growth plate, at the outer surface of tubular bones (36).

Most skeletal elements in the adult zebrafish skeleton contain osteocytes, but with a smaller volume and less canaliculi compared to mice and humans (36). The mechanosensing ability of osteocytes in zebrafish is not fully understood yet, but it was shown that osteocytes have a preferred orientation in adult zebrafish vertebrae (36). Acellular bone, without trapped osteocytes, can be found in many zebrafish cranial bones. Contrary to expectations, acellular bone does not appear to be stiffer due to the lack of osteocyte lacunae, making the role of acellular bone unclear (43). It is important to note that both cellular and acellular bone can occur within the same bony element. Osteon-like structures in zebrafish have been reported (for the lateral ethmoid bone) but these structures, composed of a central Haversian canal and bone lamella, do not have osteocytes (36).

In mammals, bone resorbing cells are multinucleated macrophages originating from the fusion and maturation of peripheral blood monocytes differentiated from hematopoietic bone marrow cells (44). Multinucleated osteoclasts can also be found in teleosts, especially in basal teleosts, such as salmonids and cyprinids (45). Nevertheless, in teleosts, smaller and mononucleated osteoclasts are predominant, but they retain the molecular regulators of mammalian osteoclast function (37). Examples include receptor activator of nuclear factor kappa-B (Rank) and Rank-ligand (Rankl) which are important for osteoclast maturation. Mature osteoclasts become tartrate-resistant acid phosphatase (Trap) and cathepsin K (CtsK) positive, which are both required for the cells to be able to degrade bone matrix components (37, 46). Zebrafish are characterized by an ontogenic change at 30 dpf when mononucleated osteoclasts evolve to multinucleated osteoclasts, which perform lacunar resorption and bone remodeling (37).

Each cell type achieves and performs its function by involving specific genes, acting as molecular fingerprints. All three bone cell types develop from similar progenitors as their mammalian counterpart and share similar profiles of gene expression (Figure 2A) (36). Gene expression of zebrafish collagen and transcription factor in skeletal cells of cartilage and bone are not completely conserved with mammals. Unlike mammals, zebrafish osteoblasts express collagen type X and various teleosts have been shown to have collagen type II in their bone matrix (47, 48). In addition, *Sox9* expression, which is required for differentiation of chondrocytes, but not of osteoblasts in mammals, has been reported to be involved in bone development in teleosts (49). Unlike tetrapods, zebrafish type I collagen, the most abundant protein in bone, has three instead of two different α chains, namely α1, α3, and α2 encoded by *col1a1a, col1a1b*, and *col1a2*, respectively (50). Based on the amino acid sequence, the α3 chain is phylogenetically similar to α1, supporting the common origin of their coding genes, which derive from a genome duplication that occurred at an early stage in teleost evolution (51). Importantly, all amino acid residues involved in human/mouse collagen type I cross-links are conserved in zebrafish, suggesting the existence of similar extracellular assembly (50).

### Bone Ossification

Bone formation starts in zebrafish around 4–5 dpf. The bony elements can have three modes of ossification: intramembranous, perichondral, or endochondral. Intramembranous ossification starts with mesenchymal cell condensation and differentiation into osteoblasts, without the need of a cartilage template (Figure 2Bi) (45). This type of ossification occurs in the skull, for example in the cranial roof and opercular bones, in the vertebral column, where most of the vertebral body is formed by this type of ossification, in scales and in the fin rays (45). In mammals, this ossification is mostly restricted to bones of the cranial vault and the dentary (52).

Perichondral ossification, characterized by bone formation in the perichondrium, is more common in the teleost compared to the mammalian skeleton, where it has been considered as a form of intramembranous ossification (45). In teleosts perichondral ossification is present in the hyomandibula and Meckel's cartilage, where osteoblasts aggregate on the surface of the cartilaginous template and deposit bone matrix into the perichondrium (Figure 2Bii).

Endochondral ossification, which is the main type of ossification in mammals, is uncommon in teleosts. In this type of ossification, mesenchymal cells condense and differentiate into chondroblasts and chondrocytes, which then secrete an extracellular cartilage matrix that functions as a template that is replaced by bone matrix (Figure 2Biii). In teleosts, two types of endochondral ossification exist. In a few bones, such as the ceratohyal and the radials in the pectoral fin, type I endochondral ossification takes place at the level of epiphysis and of the epiphysial growth plate resembling the mammalian endochondral ossification process. It is characterized by a resting zone, a proliferation zone with columnar cartilage, and a hypertrophic zone followed by a region in which cartilage matrix calcifies (36). Finally, chondroclasts degrade the cartilaginous matrix (degradation zone), allowing osteoblasts to lay down bone matrix (ossification zone). In the hyomandibula, branchial arches, ethmoid and hypuralia type II endochondral ossification takes place. Here, the calcification and ossification zones are absent and the cartilage template is replaced by adipose cells, leading to tubular concave bones filled with adipose tissue (36, 37).

Because the cranial skeleton is often too complex for screening by high throughput methods, the zebrafish vertebral body is the most investigated component of the skeleton both in early and adult life stages. Although the vertebrae in both mammals and teleosts consist of notochord and bone, there are a few key differences. First, the notochord is the *de facto* vertebral column in early teleost life stages and persists throughout life, while it only forms the intervertebral disc in mammals (53, 54). The notochord consists of a core of large and vacuolated chordocytes which is surrounded by an epithelial layer of chordoblasts that secrete the notochord sheath. This sheath is a stratified structure, composed of a thin external membrane containing elastin, covering a thicker layer of mainly collagen type II (54). Second, while the vertebrae in mammals have a cartilaginous precursor which endochondrally ossifies, zebrafish vertebrae form initially through direct mineralization of the notochord sheath, called chordacentra, in the absence of a cartilaginous precursor (55, 56). To this day, the exact cellular involvement of this notochord sheath mineralization remains unresolved. Third, the teleost vertebra is subsequently built via intramembranous ossification outside the notochord onto the chordacentrum, consisting of a compact autocentrum and trabecular arcocentrum, which forms the neural and haemal arches (56, 57). The osteoblasts produce collagen type I bone matrix and start to ossify the autocentrum at the level of the intervertebral disc, which acts as the growth center of the vertebral centrum (34).

## Generation of Knock-Out and Knock-In Zebrafish Models

### Forward Genetic Approach

Different methods to generate zebrafish models of human disorders have been explored over the last decades. Initially, a number of large-scale forward genetic screens, based on random mutagenesis with radiation, chemicals, or insertional mutagenesis, revealed zebrafish mutants affecting different aspects of embryonic development and biological processes (58–60). This phenotype-driven approach was also applied to screen for genes involved in skeletal development and diseases (Table 1). Several mutants with defects in craniofacial cartilage elements and with mineralized tissue phenotypes (119), or with changes in the shape of the skeleton (96) were identified in large scale forward genetic screens. Mapping of the causative change established some of these mutants as models for human skeletal disorders. For instance, in a study by Gistelinck et al. (120), several type I collagen zebrafish mutants, previously discovered in a forward genetic screen (96), were established as representative models for the brittle bone disorder osteogenesis imperfecta.

**Table 1:** Zebrafish models for skeletal disorders.

**Disorder**	**Gene**	**Type**	**Origin**	**References**
Acrocapitofemoral dysplasia	*Ihh*	KO	ENU	(40)
Alagille syndrome	*jagd1b*	KO	ENU	(61)
Amelogenesis imperfecta	*slc10a7*	KD	MO	(62)
Auriculocondylar syndrome	*mef2ca*	KO	ENU	(63)
Bruck syndrome	*Plod2*	KO	ENU	(16)
Campomelic dysplasia	*sox9a, sox9b*	KO	ENU	(64)
Cartilage-Hair Hypoplasia	*rmrp*	KO	CR	(65)
Cenani-Lenz syndactyly	*lrp4*	KD	MO	(66)
Chordoma	*HRASV12*	OE	Tol2	(67)
Cleidocranial dysplasia	*runx2b*	KD	MO	(68)
Craniofacial defects	*tgfb2*	KD	MO	(69)
Craniofacial defects	*fgf10a*	KD	MO	(69)
Craniosynostosis	*tcf12*		Tol2	(70)
Craniosynostosis	*cyp26b1*	KO	ENU	(71)
Craniosynostosis	*cyp26b1*	KO	ENU	(72)
Culler-jones syndrome	*gli2*	KO	Tol2	(73)
Delayed mineralization	*Pth4*			(74)
Delayed mineralization			TR	(75)
Ehlers-Danlos syndrome	*b4galt7*	KD	MO/CR	(76)
Fibrodysplasia Ossificans Progressiva	*acvr1*	CE	Tol2	(77)
Gaucher disease	*gba1*	KO	ENU	(78)
Holoprosencephaly	*ptch1*	KO	ENU	(40)
Hyperosteogeny	*n1aIcd*	OE	Tol2	(79)
Hyperthyroidism	*tshr*	KO	ENU	(80)
Hypohidrotic ectodermal dysplasia	*eda, edar*	KO	ENU	(81)
Joint disease	*scxa*	KO	CR	(82)
Klippel Feil syndrome	*meox1*		ENU	(83)
Multiple hereditary exostoses	*ext2, papst1*	KO	ENU	(84)
No mineralization	*entpd5*	KO	ENU	(85)
Oculodentodigital dysplasia	*cx43*	KO	ENU	(86)
Orofacial cleft	*tgfβ3*	KD	MO	(87)
Orofacial cleft	*mir140*	KD	MO	(88)
Orofacial cleft	*faf1*	KD	MO	(89)
Orofacial cleft	*wnt9a, irf6*	KO	Tol2	(90)
Osteoarthritis	*col11a2*	KO	ENU	(91)
Osteoarthritis	*prg4a, prg4b*	KO	TA	(92)
Osteogenesis imperfecta	*col1a1a*	MM	ENU	(14, 15, 93)
Osteogenesis imperfecta	*bmp1*	KO	ENU	(94)
Osteogenesis imperfecta	*sp7/osx*	KO	ENU	(95)
Osteogenesis imperfecta	*col1a1a, col1a1b, col1a2*	MM	ENU	(96)
Osteopetrosis	*m-csf*	KO	ENU	(97)
Osteoporosis			TR	(98)
Osteoporosis			TR	(99)
Osteoporosis	*gpr137b*	KO	CR	(100)
Osteoporosis			TR	(101)
Osteoporosis			TR	(102)
Osteoporosis	*atp6v1h*	KO	CR	(20)
Osteoporosis	*lgmn*	KO	TA	(103)
Osteoporosis	*lrp5*	KD	MO	(19)
Osteoporosis	*pls3*	KD	MO	(18)
Osteoporosis			TR	(104)
Pseudoxanthoma elasticum	*enpp1*	KO	ENU	(105)
Pseudoxanthoma elasticum	*abcc6a*	KO	ENU	(106)
Saethre-Chotzen syndrome	*twist, tfc12*	KO	TA	(107)
Saul-Wilson syndrome	*cog4*	KO	CR	(108)
Spine curvature disorders	*kif6*	KO	TA	(109)
Spine curvature disorders	*ptk7*	KO	ZFC	(110)
Spine curvature disorders	*slc39a8*	KO	CR	(111)
Spine curvature disorders	*col8a1a*	KO	ENU	(112)
Spine curvature disorders	*tbx6, her1, her7, hes6*	KO	TA	(35)
Spine curvature disorders	*uts2ra*	KO	TA	(113)
Spine curvature disorders			TR	(114)
Sponastrime dysplasia	*tonsl*	KO	CR	(115)
Stickler/Marshall syndrome	*col11a1a, col11a1b*	KD	MO	(116)
Tumoral calcinosis	*golgb1*	KO	TA	(117)
Vertebral fractures			TR	(118)

*KO, Knockout; KD, knockdown; MO, morpholino; CE, cell ablation; MM, missense mutation; ENU, N-ethyl-N-nitrosourea; CR, CRISPR; Tol2, transposon-mediated integration; TR, treatment, meaning OP models induced by microgravity, drugs, aging, physical exercise, iron stress, microRNA, mechanical loading; TA, talen; ZFN, zinc finger nuclease*.

### Reverse Genetic Approach: Morpholino Knockdown and Gene Editing

Although forward genetics brought great progress to the field of disease modeling, still, for many causal human disease genes, this approach did not reveal corresponding zebrafish mutants, as there is incomplete genome coverage of mutagenesis. Consequently, the need to investigate the function of relevant candidate genes for specific diseases or developmental pathways, sparked the expansion of reverse genetic approaches in the zebrafish field.

The assessment of candidate gene function was initially enabled via knockdown through the use of antisense morpholinos (MO). Their ease of use made this approach increasingly popular for gene function analysis, and several early studies demonstrated that MO-mediated knockdown (“morphants”) recapitulated known mutant phenotypes (121, 122). Over the past years, MOs have also been used in zebrafish modeling of skeletal disorders (Table 1). An example includes the monogenetic form of X-linked osteoporosis, caused by loss-of-function variants in *PLS3* encoding for plastin 3, a cytoskeletal protein involved in bone homeostasis. MO-mediated knockdown of *pls3* in zebrafish (18) induced malformations of the developing craniofacial bone structure, which could be reversed by the administration of human *PLS3* mRNA. Another example by Flores et al. (68) shows that depletion of *runx2b* by MO injection severely compromised craniofacial cartilage formation, phenocopying the human dominantly inherited disorder cleidocranial dysplasia, a condition characterized by impaired ossification and multiple skeletal abnormalities (68). Nevertheless, problems with the application of MOs in zebrafish emerged, such as the frequent occurrence of p53-dependent apoptosis (123–125), and off-target effects resulting in so-called “pseudophenotypes” (126, 127), but also MO-induced phenotypes that cannot be recapitulated in existing mutants (128). The latter issue has recently been studied in more detail leading to the insight that, at least for some genes, the phenotypic differences between morphants and mutants can be due to genetic compensation in the latter, but not in the former (129).

Definitive reverse genetic approaches in zebrafish recently became available in the form of site-specific nucleases enabling targeted gene modification. Initial work utilized zinc finger nucleases (ZFNs) (130, 131), and transcription activator-like effector nucleases (TALENs) (132). However, CRISPR/Cas9 genome editing is currently the most versatile and frequently employed reverse genetic technology for the creation of both knock-out and knock-in disease models. The CRISPR/Cas9 system induces a double-stranded DNA break (DSB), carried out by the Cas9 nuclease, at a specific target site, recognized by the binding of a single-guide RNA (sgRNA) molecule. Following DSB, different endogenous repair mechanisms can be initiated. On one hand, the error-prone non-homologous end joining (NHEJ) pathway can be activated, often leading to the introduction of *indel* mutations due to imprecise repair, resulting in gene knock-out. The generation of gene knock-outs in zebrafish is relatively straightforward and efficient. In a study by Zhang et al. (20) for instance, mutations in the *ATP6V1H*, coding for vacuolar ATPase, were identified in patients with short stature and osteoporosis. Loss-of-function mutants in *atp6v1h* were generated in zebrafish through CRISPR/Cas9-mediated gene knock-out (20). These mutants demonstrated loss of bone mass and increased expression of matrix metalloproteases *mmp*9 and *mmp13*. Indeed, pharmacological inhibition of mmp9 and mmp13 rescued the bone phenotype, suggesting that blockade of collagen degradation can be a valid therapeutic target. CRISPR/Cas9 gene editing has been recently used to generate knock-out zebrafish for *crtap* and *p3h1*, two genes that are part of a protein complex which is involved in prolyl 3-hydroxylation and proper folding of collagen type I. Loss-of-function mutations in the human ortholog genes cause recessive forms of OI. These zebrafish models faithfully mimic the human disease and support the defective chaperone role of the 3-hydroxylation complex as the primary cause of the skeletal phenotype (17).

In general, reverse genetic approaches are limited by the time required to generate mutant lines, where stable knock-out zebrafish are mostly obtained and analyzed from the F2 generation on. Therefore, an approach for rapid CRISPR-based reverse genetic screens was developed in which phenotyping is performed directly in F0 (mosaic) founders, which are called “crispants” (133, 134). This enables moderate to rapid throughput reverse genetic screens of candidate genes, contributing to skeletal disease. In a study by Watson et al. (133), the comparison between somatic, CRISPR-generated F0 mutants and homozygous germline mutants for *plod2* and *bmp1*, two genes implicated in recessive OI, revealed phenotypic convergence, suggesting that CRISPR screens of F0 animals may faithfully recapitulate the phenotype of skeletal disease models (133).

As an alternative to NHEJ-mediated repair of CRISPR/Cas9-induced DSB, the homology-directed repair (HDR) pathway can be initiated, but only in the presence of a homologous repair template. In physiological circumstances, HDR occurs between sister chromatids during the G2 and S phase of the cell cycle. The knock-in modeling procedure exploits this mechanism by supplying the CRISPR/Cas9 system with an artificial repair template, homologous to the target sequence and containing a specific variant of interest. For the generation of knock-in models, mostly single-stranded oligodeoxynucleotide (ssODN) repair templates are used (135) mainly because the design and production of ssODNs is easier, cheaper and results in higher HDR efficiencies compared to double-stranded templates such as plasmids (136, 137). The need to complement knock-out models with these more precise knock-in disease models is growing, for various reasons. Firstly, specific point mutations may cause a highly divergent pathobiology compared to loss-of-function mutations modeled by knock-out models. More specifically, certain missense mutations may cause a gain-of-function rather than a loss-of-function, while missense mutations in genes encoding proteins included in protein complexes may exercise a dominant negative effect and change the function of the whole protein complex. For instance, in dominant types of OI caused by mutations in the genes encoding the type I collagen α chains, depending on the type of mutation, either the quantity or the structure of type I procollagen is altered (138). The “quantitative” mutations, mostly resulting in a null *COL1A1* allele, typically cause mild forms of OI, while “qualitative or structural” defects, frequently associated with glycine substitutions, can be responsible for lethal, severe or moderate forms of the disease.

Also, missense mutations in vital developmental genes may be hypomorphic while their loss-of-function counterparts result in early lethality, as reported in the *cdc6* zebrafish mutant for Meier-Gorlin syndrome. Hypomorphic mutations in the *cdc6* gene recapitulate the patient's phenotype, while the knock-out mutants are embryonically lethal. In these cases, the introduction of such point mutations is a prerequisite to faithfully recapitulating human disease. Secondly, as mentioned before, several zebrafish knock-out models failed to generate a phenotype, which can be due to mRNA decay-induced genetic compensation (139), a phenomenon that is not expected to occur in knock-in models.

Nevertheless, several drawbacks mitigate the straightforward use of HDR knock-in zebrafish models. Firstly, HDR pathways have proved highly inefficient for genome editing (140) even despite proposed modifications, such as repair template modification (141, 142), cell cycle arrest (143) and chemical compound administration (144–151). Secondly, CRISPR/Cas9-mediated HDR mechanisms have been shown to be error-prone (152, 153). These issues hindered the development of knock-in zebrafish models and only a limited number have been reported, in contrast to numerous knock-outs. For instance, CRISPR/Cas9-mediated point mutation knock-ins have been generated for genetic variants implicated in inherited cardiac diseases (154–156), although to our knowledge none have been described so far for skeletal diseases. Different recently developed DSB-free alternatives for precise base pair substitution, such as programmable base editing (157–159) and prime editing (160) promise to be more efficient and versatile approaches, but more research is needed to further improve these methods for application to the zebrafish model system.

## Transgenic Lines

### Transgenic Zebrafish to Trace Bone Cells and Pathways

Despite the development of new approaches in large-scale and more recently single-cell transcriptomics, genomics, epigenomics, and proteomics (161), these techniques are time consuming, expensive and only available in specialized laboratories (162–164). Furthermore, retrospective -omic analyses exclude cells that do not survive to the point of cell harvest, a common and necessary event in growth and regeneration. Therefore, to be able to understand the dynamic nature of tissue development and regeneration, *in vivo* time-lapse imaging is essential.

The recent evolution of genetic engineering has allowed the generation of transgenic animal models, expressing fluorescent proteins under cell- or pathway- specific promoters, enabling *in vivo* imaging of differentiation and signaling (165). However, the generation of transgenic murine models remains technically demanding, time consuming and expensive (166). In addition, since mice develop *in utero*, it is almost impossible to investigate early developmental processes in real time and the visualization at cellular level usually requires post-mortem analyses (167).

Zebrafish, with its fast external development, transparent early life stages and relative easy genetic manipulation, is rapidly becoming the model of choice for examining developmental processes via time-lapse microscopy. The introduction of reporter genes downstream of a specific promoter makes it possible to produce site-directed indicators in different organs, tissues or cells and permits real time imaging in developing embryos or post-hatch stages; or even in mature zebrafish by fluorescent microscopy on whole mount specimens (168, 169). A variety of transgenic reporter lines have been generated to mark skeletal cell lineages at different stages of differentiation and signal transduction pathways, by using the conserved regulators of skeletal development (Table 2). The availability of fluorescent reporter lines, together with the use of powerful techniques such as two or multi-photon or light sheet microscopy, has allowed imaging of tissues and organs at a cellular and subcellular level, especially by exploiting the transparency of early life stages (218).

**Table 2:** Transgenic lines employed to study zebrafish skeleton.

**Cell type**	**Gene/pathway**	**Transgenic line**	**References**	**Applications**
Neural crest-derived skeletal cells	*sox10*	Tg*(sox10:GFP)^*ba5*^*	(170)	(170)^#^, (19)^*^
	*sox10*	Tg*(sox10:kaede)^*zf393*^*	(171)	(90, 171)^#^
	*sox10*	Tg*(sox10:mRFP)^*vu234*^*	(172)	(78, 172)^*^
	*sox10*	Tg*(-4725sox10:Cre)^*ba74*^*	(173)	(173, 174)^#^
	*sox10*	Tg*(−4.9sox10:egfp)^*ba2*^*	(175)	(175–177)^#^
	*fli1*	Tg*(fli1:EGFP)^*y1*^*	(178)	(19, 78, 89, 178, 179)^*^
Cartilaginous cells	*foxp2*	Tg(*foxp2-enhancerA:EGFP*)*^*zc42*^*	(180)	(180, 181)^#^
	*col2a1a*	Tg(*Col2a1aBAC:mcherry*)*^*hu5910*^*	(40)	(78, 91, 105)^*^, (40, 182)^#^, (76)^*^
	*col2a1a*	Tg*(-1.7col2a1a:EGFP-CAAX)^*nu12*^*	(183)	(183, 184)^#^, (112)^*^
	*col18a1*	Tg*(16Hsa.COL18A1-Mmu.Fos:EGFP)^*zf215*^*	(185)	(185)^#^
Preosteoblasts	*cyp26b1*	Tg*(cyp26b1:YFP)^*hu5786*^*	(72)	(72)^#^
	*cyp26b1*	*Tg(cyp26b1:YFP)^*hu7426*^*	(186)	(186)^#^
Branchial arches and notochord cells	*cyp26a1*	Tg*(cyp26a1:eYFP)^*nju*1/+^*	(187)	(187, 188)^#^
Intervertebral disc cells	*shhb*	Tg*(-5.2shhb:GFP)^*mb*1^*	(189)	(189)^#^
	*twist*	Tg*(Ola.twist1:EGFP)^*ca104*^*	(190)	(190)^#^
Early osteoblasts	*osx/sp7*	Tg*(sp7:EGFP)^*b1212*^*	(181)	(73, 181)^#^, (112, 179, 191, 192)^*^, (193)^§^, (65)^*^
	*osx/sp7*	Tg*(Ola.sp7:mCherry)^*zf131*^*	(72)	(94)^*^, (72)^#^
	*osx/sp7*	Tg *(Ola.sp7:NLS-GFP)^*zf132*^*	(72)	(194)^§^, (72, 195)^#^, (78, 85)^*^, (196)^#^
	*osx/sp7*	Tg*(osterix:mCherry-NTRo)^*pd46*^*	(197)	(197, 198)^§^
	*osx/sp7*	Tg*(osx:Kaede)^*pd64*^*	(198)	(196, 199)^#^, (198)§
	*osx/sp7*	**Tg*****(osx:CFP-NTR)***	(200)	(200)^#^
	*osx/sp7*	Tg*(osx:H2A-mCherry)^*pd310*^*	(198)	(198)^§^
	*osx/sp7*	Tg*(osterix:Lifeact-mCherry)^°*u2032*^*	(201)	(201)^§^
	*col10a1*	Tg*(Col10a1BAC:mCitrine)^*hu7050*^*	(202)	(78, 91, 105)^*^, (202)^#^
	*col10a1*	Tg*(-2.2col10a1a:GFP)^*ck*3^*	(203)	(203, 204)^#^
	*runx2*	Tg*(Hsa.RUNX2-Mmu.Fos:EGFP)^*zf259*^*	(205)	(95, 195)^#^, (205)^§^
	*runx2*	Tg*(RUNX2:egfp)*	(31)	(31)^#^, (182)^*^
Mature osteoblasts	*osc/bglap*	Tg(*Ola.bglap.1:EGFP*)*^*hu4008*^*	(205)	(105, 195)^*^, (205)^§^
	*entpd5a*	Tg*BAC(entpd5a:YFP)^*hu5939*^*	(85)	(35)^#^, (85)^*^
	*entpd5a*	*TgBAC(entpd5a:Kaede)^*hu6867*^*	(195)	(195)^*^, (35)^#^
	*col1a1*	Tg*(col1a1:EGFP)^*zf195*^*	(31)	(31)^#^, (18)^*^
	*rankl*	**Tg*****(rankl:HSE:CFP)***	(46)	(46)^*^
	*notch1a*	Tg*(Ola.sp7:N1aICD)^*cy31*^*	(79)	(79)^#^
Osteoclasts	*ctsk*	Tg*BAC(ctsk:Citrine)^*zf336*^*	(206)	(105)^*^
	*ctsk*	Tg*(ctsk:YFP)*	(206)	(105)^*^
	*ctsk*	Tg*(ctsk:DsRed)*	(207)	(207)^#^
	*ctsk*	**Tg*****(CTSK-DsRed)***	(97)	(97)^#^
	*ctsk*	Tg*(Ola.ctsk:EGFP)^*zf305*^*	(97)	(97)^#^
	*ctsk*	**Tg*****(ctsk:mEGFP)***	(46)	(46, 208)^*^
	*trap*	**Tg*****(TRAP:GFP)***	(97)	(97)^#^
	*trap*	Tg*(trap:GFP-CAAX)^°*u2031*^*	(201)	(201)^§^
Bmp responsive cells	Bmp pathway	Tg*(Bre:GFP)^*p77*^*	(209)	(209)^#^
	Bmp pathway	Tg*(bre:egfp)^*pt510*^*	(210)	(177, 210)^#^
	Bmp pathway	Tg*(BMPRE:EGFP)^*ia18*^*	(169)	(169)^#^, (78)^*^
β-catenin activated cells	Wnt pathway	Tg*(7xTCF-Xla.Siam:GFP)^*i*a*4*^*	(211)	(211)^#^, (78)^*^
	Wnt pathway	Tg*(7xTCFXla.Siam:nlsmCherry)^*ia5*^*	(211)	(73, 211)^#^
	Wnt pathway	Tg(*hsp70l:wnt8a*-*GFP*)*^*w34*^*	(212)	(213)^#^
	Wnt pathway	Tg(*hsp70l:dkk1-GFP*)^w32^	(214)	(73)^#^, (214)^§^
	Wnt pathway	Tg(*myl7:EGFP*)*^*twu34*^*	(215)	
Stress responsive cells	UPR pathway	Tg*(ef1α:xbp1δ-gfp)^*mb10*^*	(216)	(216)^#^
	UPR pathway	Tg(Hsa*.ATF6RE:*d2GFP*)^*mw85*^*	(217)	(217)
	UPR pathway	Tg*(*Hsa*.ATF6RE:*eGFP*)^*mw84*^*	(217)	(217)

* *Transgenic lines used to characterize mutants with skeletal pathologies*,

# *transgenic lines used to analyse skeletal development and molecular pathways*,

§ *transgenic lines used to study skeletal regeneration, Medaka transgenic lines are reported in bold*.

### Transgenic Lines to Trace Bone Cells

The most frequently used lines expressing fluorophores in chondrocytes include Tg*(*−*4.9sox10:egfp)*^*ba*2^ and Tg*(Col2a1aBAC:mcherry)*^*hu*5910^ (Table 2). The Tg*(*−*4.9sox10:egfp)*^*ba*2^ was employed to detect *sox10* expression in head cartilage during embryo development and to follow migration of neural crest cells during cranium morphogenesis (175). The Tg*(Col2a1aBAC:mcherry)*^*hu*5910^ reporter line allowed impaired cartilage patterning and loss of chondrocyte organization to be shown in a zebrafish model of a recessive form of Ehlers-Danlos syndrome with partial loss of B4galt7, a transmembrane Golgi enzyme that plays a pivotal role in proteoglycan biosynthesis (76).

In order to trace the differentiation of bone forming cells, transgenic lines for both early and late osteoblast markers, expressing fluorophores under the *osterix/sp7* and *osteocalcin*/*bglap* promoters, have been generated (Table 2). The Tg*(sp7:EGFP)*^*b*1212^ line allowed osteoblast behavior to be studied during both intramembranous and endochondral ossification. Moreover, this line was used to investigate the abnormal perichondral ossification in the RNA component of the mitochondrial RNA-processing endoribonuclease (*rmrp*) knock-out zebrafish model of cartilage hair hypoplasia (65). *Tg(Ola.sp7*:*mCherry)*^*zf*131^ was crossed with the OI type XIII zebrafish model *frilly fins* to elucidate the role of the bone morphogenic protein 1, encoded by *bmp1a* gene, in osteoblast differentiation and localization (94).

The Tg*(Ola.bglap.1:EGFP)*^*hu*4008^ line was used to understand the fundamental role of osteoblast dedifferentiation during bone healing in response to traumatic injury, and to show that adult zebrafish osteoblasts display an elevated cellular plasticity compared to their mammalian counterpart (195).

Despite the conservation of most of the osteoblastogenic markers, in zebrafish the expression of *col10a* is not limited to chondrocytes as in mammals, but is also present in osteoblasts (203). The transgenic line Tg*(-2.2col10a1a:GFP)*^*ck*3^, expressing GFP under *col10a1* promoter, has therefore been used to investigate molecular events driving both chondrocyte and osteoblast development (203).

An interesting application of the transgenic reporter lines is their use in combination with a mineral stain, imaged at different fluorescent wavelengths, enabling the combined study of osteoblast dynamics and bone mineralization (196). For instance, alizarin red staining of the transgenic zebrafish Tg*(Ola.sp7:NLS-GFP)*^*zf*132^ localized *osterix/sp7* positive osteoblasts in the mineralized bone and revealed the absence of *osterix/sp7* expression in the anterior notochord region at 8 dpf (104). Similarly, mineral staining in combination with Tg(*osx*:Kaede)^pd64^ confirmed the osteoblast independent mineralisation of the notochord (196).

Most of the available osteoclast reporter lines express fluorophores under control of the promoter of cathepsin K (Ctsk), the osteoclast collagenase that mediates bone resorption (Table 2) (46). Chatani et al. (97) proved the absence of osteoclasts in the *panther* mutant, which lacks a functional receptor for the macrophage colony stimulator factor, taking advantage of the Tg*(ctsk:mEGFP)* transgenic line. A significantly reduced number of GFP-positive osteoclasts was found in the neural and haemal arches in *panther* larvae, indicating a crucial role of the protein in osteoclast proliferation and differentiation. Additionally, the medaka, another well-characterized teleost bony fish used for developmental and biomedical studies, was used to study osteoclasts by placing the gene encoding for the receptor activator of nuclear factor kappa-B ligand, *rankl*, a key osteoclast differentiation factor, under the control of a heat shock element (23). Increased osteoclast differentiation induced upon Rankl activation in this Tg*(rankl:HSE:CFP)* line resulted in an osteoporotic phenotype (46).

### Transgenic Lines to Trace Signal Transduction Pathways

Zebrafish transgenic lines expressing *in vivo* reporter proteins under the control of signaling pathway responsive elements are a powerful tool to dissect dynamically the *in vivo* activation or repression of endogenous signaling pathways in real time (210, 219–221). Calcium, Bmp and Wnt pathways are crucial players during bone formation (222–224). Transgenic lines to further investigate these pathways have been generated (Table 2). The Tg*(hsp70:bmp2b-GFP)* line was used to analyze the role of the Bmp2 signaling pathway in an enteric disease, but the transgenic model could be employed to dissect BMP2b signaling in bone (225). To investigate Wnt pathway activation the Tg*(7xTCF-Xla.Siam:GFP)*^*ia*4^ and Tg*(7xTCF-Xla.Siam:nlsmCherry)*^*ia*5^ transgenic lines, which contain multimerized *tcf/lef* binding sites for the transcription factor activated by β*-catenin* upstream to a siamois minimal promoter, were generated allowing the dynamics of neural crest-derived cell migration to be traced during development (211). Using the Tg*(7xTCF-Xla.Siam:nlsmCherry)*^*ia*5^ transgenic line it was also possible to elucidate important regulatory steps in the osteogenic differentiation process of mesenchymal stem cells (73).

Finally, the unfolded protein response (UPR) was shown to play an important role in the modulation of the phenotype in rare skeletal diseases (226, 227). Interestingly, transgenic zebrafish lines allowing different branches of this pathway to be followed are already available (216, 217, 228, 229). For instance, the transgenic zebrafish model Tg*(ef1*α*:xbp1*δ*-gfp)*^mb10^ has been used to trace *in vivo* the splicing of *xbp1*, one of the terminal effectors of the UPR (216).

### Live Imaging of Bone Regeneration

Tracing bone cells *in vivo* using transgenic lines in adult zebrafish is challenging due to tissue depth and complexity, but is possible in external structures such as fin rays or scales, which are easily accessible and suitable for regeneration studies (198, 230, 231). Indeed, the available panel of transgenic lines expressing fluorescent and photo-switchable reporter genes in bone cells is useful to trace regeneration *in vivo* (198). This strategy has clarified important biological aspects such as the cellular basis of integumentary bone regeneration. *In vivo* imaging of the Tg*(sp7:EGFP)*^*b*1212^ transgenic line during caudal fin regeneration showed the presence of GFP positive cells at the amputation plane starting from 2 days post amputation (dpa) and their association with the formation of newly mineralized matrix by 5 dpa (181). Osteoblast lineage tracing in the Tg*(osx:Kaede)*^*pd*64^ clarified migration and dedifferentiation of scleroblasts during fin regeneration (196).

However, the slow rates of regeneration require long-term live imaging to capture dynamic cellular events to improve the understanding of development, homeostasis, and regeneration by stem cell populations (232). Thus, to enable up to 24 h of continuous live imaging, specific protocols for long-term anesthesia of adult zebrafish have been optimized (198). Indeed, the transgenic line Tg*(osx:H2A-mCherry)*^*pd*310^ allowed spatio-temporally distinct cell division, motility, and death dynamic within a founder osteoblast pool to be imaged as bone regenerates (198).

### Transgenic Lines as Tool for Drug Screening

Transgenesis is not only used to analyze bone development over time, to assess a mutant phenotype or track cell signaling, but also to evaluate drug screening effects (98, 104). Huang and colleagues employed the transgenic line Tg(O*la.sp7:NLS-GFP*)^zf132^ to test anti-osteoporosis chemical drugs. This line, that expresses GFP under control of *osterix/sp7*, allowed for a faster *in vivo* evaluation of drug effects on bone mass and density compared to traditional staining methods. In another study, the *osteocalcin/bglap* reporter transgenic line Tg*(Ola.Bglap:EGFP)*^*hu*4008^ was employed to test chlorpropamide effects on the nuclear factor kappa-light-chain-enhancer of activated B cells (NF-kB). The drug negatively regulated osteoblast-like cell dedifferentiation, thus helping to maintain bone forming cells in an active state promoting caudal fin ray regeneration (233).

### Tips for Transgenic Lines Selection

For the proper selection of transgenic lines there are some aspects that require consideration. First, the choice of the reporter protein is influenced by differences such as color, brightness, toxicity, tissue penetration, subcellular localization, as well as the stability of the fluorescent protein. For instance, in order to study cell signaling dynamics or when performing prolonged cell lineage tracing, the use of long half-life fluorescent proteins is recommended. Furthermore, differences in signal pattern and intensity can be found among transgenic progeny possibly due to multiple insertions in the same founder, thus complicating the analysis (169). This aspect can be ameliorated by diluting the number of transgenic copies through subsequent generations.

Finally, in order to verify the localization of the reporter protein, the use of dual color analysis in the same transgenic line is recommended (196, 199) by for example complementary secondary techniques such as immunohistochemistry or *in situ* hybridization (169, 199).

## X-Ray Imaging

One of the more frequently used techniques to visualize the human skeleton is x-ray imaging. Classic x-ray systems for human and veterinary purposes need to limit radiation exposure to the patient, and therefore have limited exposure settings, that is their range of tube accelerating voltage (kV), current (mA), and time of exposure. These parameters are set to optimize the image of the skeleton while keeping the radiation exposure to the patient as low as possible and cannot be easily changed. Consequently, these medical appliances are not appropriate to image the small zebrafish skeletons. Examples of x-ray sources that have a wide range of possible x-ray output settings are small manual units used to scan museum artifacts and fossils, a small animal radiation research platform (SARRP; Xstrahl, Surrey, UK) and the Faxitron^©^ x-ray cabinets. Specifically, these sources can be set to low power but long exposure time parameters, and can be used in combination with high resolution technical film such as mammography film or x-ray film (e.g., AGFA D2) used in aerospace and petroleum factory applications. A Faxitron x-ray cabinet in combination with mammography film was used by Fisher et al. (93) to image the skeleton of WT and *chihuahua* mutant zebrafish to screen for skeletal abnormalities (Table 2).

**Figure 3:**
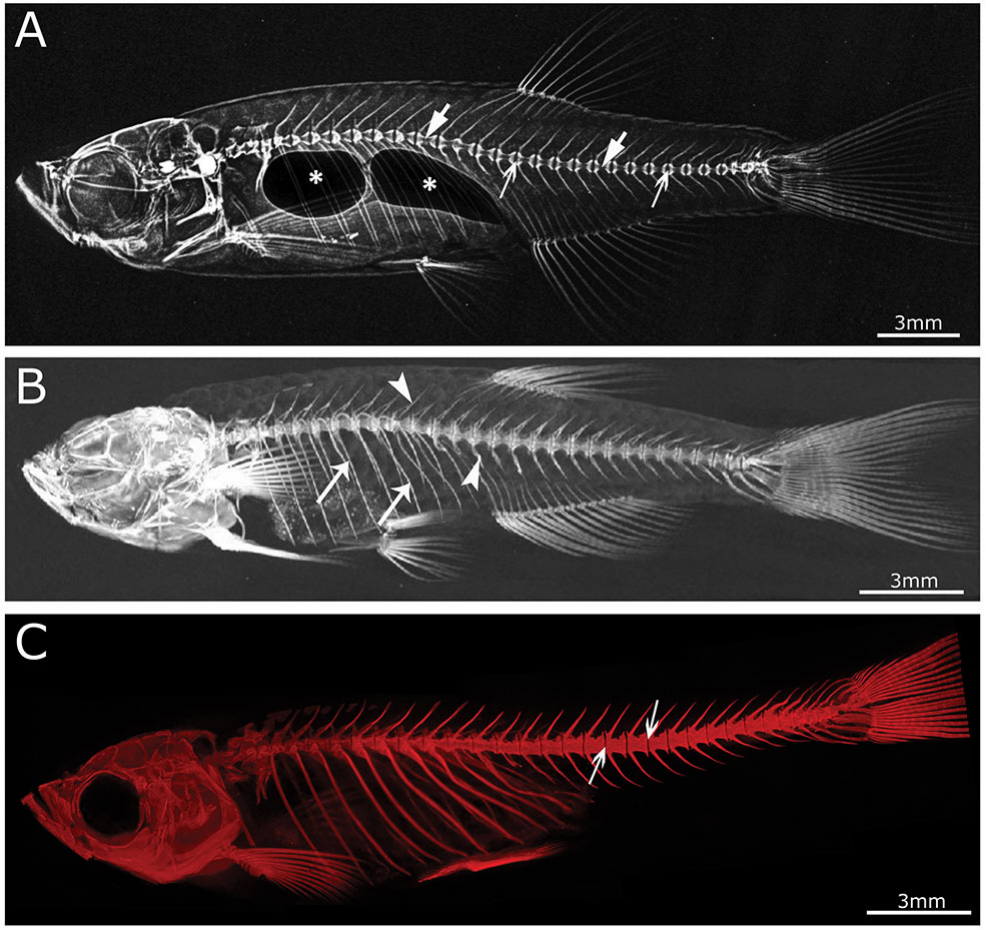
Imaging techniques in zebrafish. **(A)** Lateral x-ray image of a wild type zebrafish acquired with a Faxitron tabletop X-ray cabinet. Notice the outline of the major bones in the skull and vertebral column and the outline of the double chambered swim bladder (indicated by asterisks) in the abdominal cavity. The tissue inside the vertebrae (indicated by block arrows) and intervertebral spaces (indicated by line arrows), i.e., the notochord, can be easily assessed for the presence of mineral. **(B)** Lateral view of a 3D reconstructed microCT scanned adult zebrafish at 21 μm. More details are visible in the skull and especially the vertebral column compared to the x-ray image (neural and haemal arch are indicated by arrow heads and the ribs with a small arrows). **(C)** Lateral image in the fluorescent channel of a zebrafish whole mount cleared and stained with alizarin red for mineralized tissues. Compared to the images above, more details of the skeleton can be observed, especially in the vertebral column where all individual bones and their outlines can be noticed. The alizarin red image also allows to assess the presence of mineral in the intervertebral space (indicated by arrows). All images were taken of wild type zebrafish.

With the revolution of digital sensors capturing the x-ray signal, it has become straightforward to take an x-ray image of a small or large part of the human skeleton. The use of digital x-ray sensors is however more challenging when using zebrafish (24, 234) as the resolution is too low in most cases to capture a quality image of the small zebrafish skeleton. A modern system such as a Faxitron Ultrafocus x-ray cabinet can provide digital x-ray images up to a 5 μm spatial resolution which can be geometrically magnified (Faxitron^©^) (Figure 3A). This technique was used to screen for deformed and fragile bones in *chihuahua* mutant zebrafish (15) and to assess the gross skeletal anatomy of *prg4a*^−/−^*; prg4b*^−/−^ mutant zebrafish (92). Although these digital images may look clean and sharp, the thinner less mineralized bones may not be present in the image, which represents a loss of information about the zebrafish skeleton (234). In contrast, technical film such as AGFA D2 can theoretically capture extremely high-resolution images. Such technical film works well in combination with low energy settings needed for optimal imaging of the zebrafish skeleton. Moreover, this film is able to capture an image of smaller bones, which is not always possible when using a digital sensor.

The main advantage of using x-rays to image the zebrafish skeleton is that it is a cheap and quick methodology. Furthermore, x-ray imaging can be repeated on live organisms and can be used as a preliminary diagnostic tool for skeletal imaging before applying a more specialized method such as micro computed tomography (microCT) or mineral staining (Figures 3B,C). For instance, x-ray imaging is frequently used in aquaculture related research where it is a first line tool to assess skeletal deformities (235, 236). Although x-ray imaging can be employed to assess skeletal deformities in adult zebrafish, its use for juvenile zebrafish, where the skeleton is too small to be captured on film or digitally, is not feasible. In addition, x-ray images of zebrafish are not suitable for quantification of tissue or bone mineral densities. MicroCT currently provides a better solution to estimate these bone parameters (80, 120).

## Micro Computed Tomography

Computed tomography (CT) is a non-invasive technology based on x-ray analysis that allows detailed 3D reconstructions of large specimens. The generation of CT images involves the capturing and recording of x-rays that pass through the sample onto a detector. This process is repeated several times for multiple angles, followed by the virtual reconstruction into a 3D image (237). The required resolution for zebrafish imaging is beyond the capabilities of medical CT machines (≥70 μm), requiring higher resolutions, which can be obtained by microCT (Figure 3B) (237). The resolutions that can be achieved with modern microCT scanners vary from relatively low resolutions (≥20 μm), with quick scan times and large sample size, to higher resolutions (≤10 μm), with longer scanning durations and smaller sample size. It is important to note that the magnification, often described as the size of the voxels (3D pixels) is not identical to spatial resolution, which is roughly 2–3 times larger (238). MicroCT is less time consuming and provides excellent 3D resolution compared to optical microscopy/histology. Although mainly mineralized tissues are recorded, resulting in a loss of information on aspects such as cells and non-mineralized tissues, the use of contrast agents allows visualization of different tissues such as adipose or epithelial tissue and can even enhance the signal of poorly mineralized bone (239, 240). For example, scanning of juvenile stages can be performed by staining the samples with silver nitrate beforehand, allowing for visualization of early bone development where only low amounts of mineral are present (241). However, with this approach only relative mineralization densities can be determined, and not absolute hydroxyapatite levels, which is an important parameter when modeling skeletal disorders. The amount of hydroxyapatite present in samples can be determined by performing a calibration microCT scan of a reference object (phantom) with a known hydroxyapatite concentration. This approach was used in a study of the effect of aging on bone mineral density (BMD) in zebrafish, revealing progressively increased BMD with age, in contrast to humans (101). When interpreting skeletal phenotypes, it is important not to rely on a single method, because certain phenotypes can be better detected using other methods. For example, a mineralized notochord leading to completely solid centra is easier to assess using microCT compared to mineral staining (72). In addition to 3D renderings, microCT data allows the creation and viewing of individual slices throughout the sample, similar to histological sections. Histology of mineralized tissues is notoriously difficult and requires special protocols because samples cannot be demineralized for sectioning. As an example, a complementary approach of both histology and high resolution microCT (6 μm) was used in a zebrafish model for craniosynostosis revealing fusion of the coronal suture (107).

**Figure 4:**
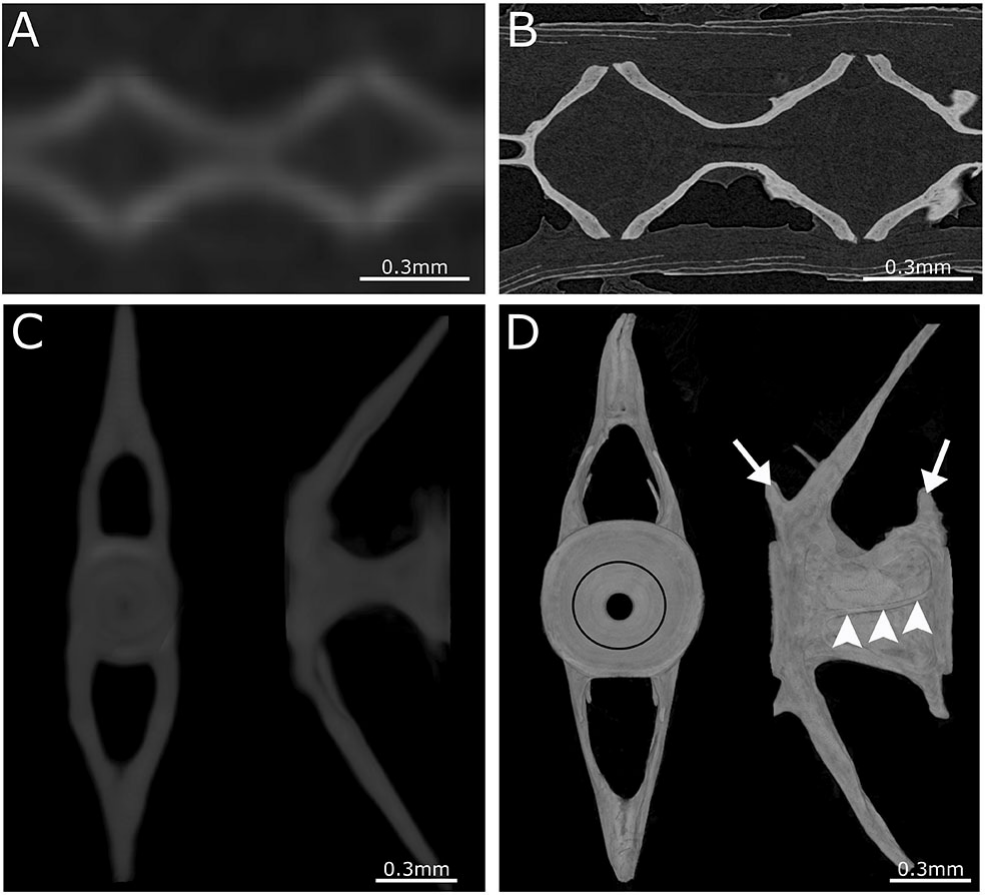
Comparison between low- and high-resolution microCT. **(A)** Image of parasagittal microCT plane at 21 μm. **(B)** Similar structure as in **(A)** but scanned at 0.75 μm. Comparison between low-resolution and high-resolution microCT clearly demonstrates the ability to distinguish separate vertebrae and compact bone only using high-resolution microCT. **(C)** Anterior and lateral view of a 3D maximal projection surface render of a vertebrae scanned at 21 μm. **(D)** Similar structure as in **(C)** but scanned at 0.75 μm. Notice the difference in detail where the growth rings (black circle) are visible in the vertebral endplate on the anterior view. The lateral view of high-resolution microCT shows the outline of the vertebra with the pre- and post-zygapophyses (white arrows), and an antero-posterior running medial vertebral trabecula (white arrowheads).

Although low resolution microCT (≥20 μm) does not allow the detection of subtle skeletal changes, such as fusions between adjacent bones, it is perfectly suitable for whole-body scanning and phenotyping of adult zebrafish with a moderate throughput (Figure 3B). Such a procedure was applied by Gistelink et al. (120), where individual vertebral bodies (neural/haemal arches and centrum) of different OI zebrafish models were manually segmented. Subsequently, tissue mineral density (TMD), vertebral length, bone volume, and thickness were determined for each component (80). Manual segmentation is a laborious process and possibly introduces human bias into the analysis, which can be overcome by semi-automated segmentation algorithms such as FishCut (80). FishCut enables the measuring of a large number of parameters in the vertebral column, and is supplemented by a statistical approach for analysis (80). Models for Bruck syndrome, osteogenesis imperfecta and hyperthyroidism have been successfully analyzed by this high-throughput pipeline, thereby standardizing zebrafish skeletal analyses (80, 120). High resolution microCT (≤10 μm) on the other hand, allows for more detailed analysis, but is very time consuming and limits the scanning to only small segments of the skeleton (Figure 4). MicroCT scans of a vertebral body at 1 μm voxel size revealed osteocyte lacunae, which is beyond the resolution range of whole body microCT scans (Figures 4B,D) (242). In a study by Newham et al. (118), high resolution scans of vertebral bodies before and after mechanical compression were analyzed via geometric morphometrics. The obtained measurements were successfully used to determine the deformation zones and subsequently used to predict the deformation and strain during loading (118).

## Bone Histology: From Whole Mount to Sections

Whole mount staining and high-resolution section analysis of the zebrafish skeleton represent complementary techniques, commonly used to describe bone development and structure at tissue and cellular levels.

### Whole Mount Mineral and Cartilage Staining

**Figure 5:**
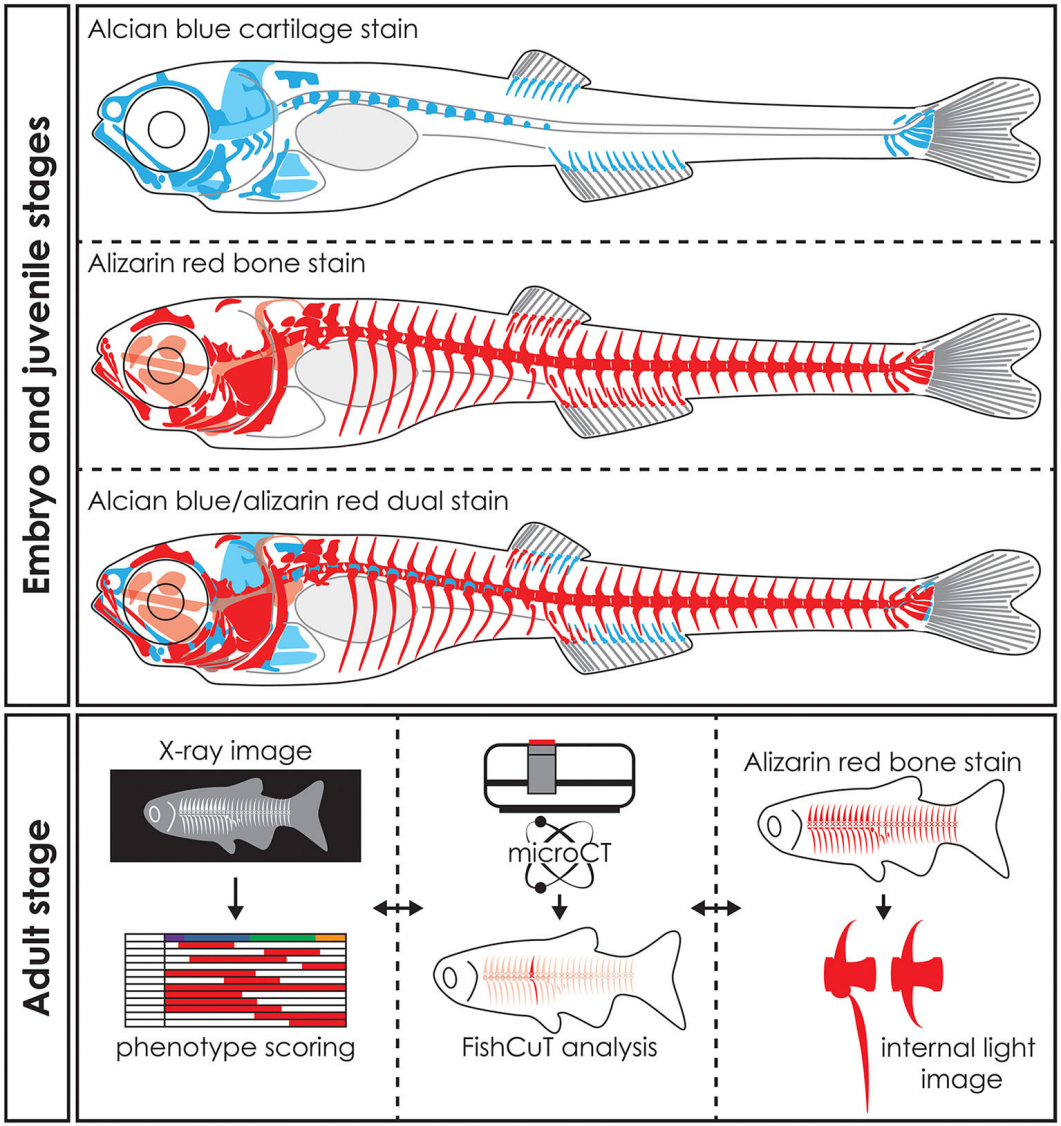
Whole mount staining in early stages and applications of visualization techniques in adult zebrafish. Schematic representation of whole mount cleared and stained early stage zebrafish for cartilage with alcian blue, mineralized tissues (bone) with alizarin red and dual stained for both cartilage and mineralized tissues. Notice that only part of the skull, the basiventrals [for definition see Gadow and Abbott (243)] of the abdominal vertebrae and the fins endoskeleton are pre-formed in cartilage. Many bones in the skull and especially in the vertebral column are formed by direct intramembranous ossification. Images of adult skeletons taken by x-ray can be used to score for skeletal abnormalities, while microCT data can be used in an analysis program such as FishCuT to obtain quantitative data of bone measurements such as size, volume, thickness, and bone mineral density (80, 120). Bright field images or fluorescent images of whole mount cleared and stained zebrafish for mineralized tissues with alizarin red can be used to study skeletal abnormalities in detail. The three techniques are mostly used on euthanized and fixed specimens and thus can be applied on the same specimen sequentially. Moreover, the data procured by these visualization techniques can be integrated into a large data matrix and allows detailed phenotypic descriptions of zebrafish disease models.

In biomedical research, where the zebrafish is used as a model organism, whole mount staining is generally used to study the morphology of the skeleton (Table 3). The most commonly used techniques are staining of mineralized tissues with alizarin red S (ARS), staining of cartilage matrix with alcian blue (AB) or staining both tissues with a combination of both ARS and AB (Figure 5). These staining methods are based on well-established protocols, where a specimen is made translucent to transparent and cartilage matrix or mineralized tissues are stained with a dye. Images of whole mount cleared and stained animals, taken with a modern stereo microscope, have an even higher resolution than standard microCT images (Figures 3B,C). Therefore, the whole mount clearing and staining technique can be considered as the gold standard for observing the whole zebrafish skeleton in detail.

**Table 3:** Techniques applied to evaluate bone phenotype in zebrafish models.

**Disorder**	**Stage**	**AR**	**AB**	**Dual stain**	**Calcein**	**Morphology**	**Histology**	**TEM**	**SEM**	**ISH**	**Transgenics**	**MicroCT**	**X-Ray**	**AFM**	**qBei**	**Nanoindentation**	**FTIR**	**References**
Acrocapitofemoral dysplasia	L			x							x							(40)
Alagille syndrome	L			x	x					x								(61)
Amelogenesis imperfecta	L	x	x															(62)
Auriculocondylar syndrome	L			x		x												(63)
Bruck syndrome	L-J-A	x	x			x	x	x				x						(16)
Campomelic dysplasia	L		x		x	x				x								(64)
Cartilage-Hair Hypoplasia	L	x		x		x				x	x							(65)
Cenani-Lenz syndactyly	L		x			x				x								(66)
Chordoma	L						x	x			x							(67)
Cleidocranial dysplasia	L		x				x			x								(68)
Craniofacial defects	L			x			x											(69)
Craniofacial defects	L			x			x											(69)
Craniosynostosis	L-A	x								x	x							(70)
Craniosynostosis	L			x		x				x								(71)
Craniosynostosis	L-A	x		x	x	x					x	x						(72)
Culler-jones syndrome	A				x		x				x							(73)
Delayed mineralization	L			x			x			x	x							(74)
Delayed mineralization	L-A	x		x		x												(75)
Ehlers-Danlos syndrome	L	x	x			x												(76)
Fibrodysplasia ossificans progressiva	L-A	x				x	x					x						(77)
Gaucher disease	L			x		x		x			x							(78)
Holoprosencephaly	L			x							x							(40)
Hyperosteogeny	L-A			x	x	x	x				x	x						(79)
Hyperthyroidism	A											x						(80)
Hypohidrotic ectodermal dysplasia	A	x		x		x					x	x						(81)
Joint disease	L-A	x		x		x	x			x	x	x						(82)
Klippel Feil syndrome	L A	x																(83)
Multiple hereditary exostoses	L	x	x							x								(84)
No mineralization	L-A	x	x	x		x				x	x							(85)
Oculodentodigital dysplasia	A								x							x		(86)
Orofacial cleft	L	x	x	x		x												(87)
Orofacial cleft	L			x														(88)
Orofacial cleft	L		x			x				x	x							(89)
Orofacial cleft	L		x			x					x							(90)
Osteoarthritis	L-A			x			x				x	x		x				(91)
Osteoarthritis	L-A		x				x			x	x	x	x					(92)
Osteogenesis imperfecta	L-A			x						x			x					(93)
Osteogenesis imperfecta	L-A	x		x		x	x			x	x							(94)
Osteogenesis imperfecta	L-A	x					x	x		x	x							(95)
Osteogenesis imperfecta	L-A	x				x						x						(96)
Osteogenesis imperfecta	L-A			x	x	x						x	x					(15)
Osteogenesis imperfecta	L-A			x	x							x			x	x	x	(14)
Osteopetrosis	L-A	x			x	x	x	x										(97)
Osteoporosis	L				x	x												(98)
Osteoporosis	L	x																(99)
Osteoporosis	A					x	x					x						(100)
Osteoporosis	A					x						x						(101)
Osteoporosis	L	x	x	x	x						x							(102)
Osteoporosis	L-A	x	x	x	x	x						x						(20)
Osteoporosis	L	x				x												(103)
Osteoporosis	L			x		x	x				x							(19)
Osteoporosis	L										x							(18)
Osteoporosis	L	x				x					x							(104)
Pseudoxanthoma elasticum	L-J	x		x	x	x					x							(105)
Pseudoxanthoma elasticum	L-J	x				x				x	x							(106)
Saethre-Chotzen syndrome	A	x		x				x				x						(107)
Saul-Wilson Syndrome	L		x															(108)
Spine curvature disorders	L-J-A			x		x				x		x						(109)
Spine curvature disorders	L-J-A				x	x						x						(110)
Spine curvature disorders	J-A	x																(111)
Spine curvature disorders	L-A	x	x			x		x			x	x						(112)
Spine curvature disorders	L-A	x			x					x	x							(35)
Spine curvature disorders	L-A					x				x		x						(113)
Spine curvature disorders	A					x	x					x						(114)
Sponastrime dysplasia	L			x														(115)
Stickler/Marshall syndrome	L		x			x	x	x										(116)
Tumoral calcinosis	A								x		x							(117)
Vertebral fractures	A					x						x						(118)

*L, Larval stage; J, Juvenile stage; A, Adult stage; AR, Alizarin red; AB, Alcian blue; TEM, Transmission electron microscopy; SEM, Scanning electron microscopy; AFM, Atomic force microscopy; qBei, Quantitative backscattered electron imaging; FTIR, Fourier-transform infrared spectroscopy*.

#### Alizarin Red S

Many different protocols exist for ARS staining of mineralized tissues, however the main steps are based on (i) removing the pigmentation of the tissue with a bleaching solution (basic pH), (ii) neutralization of depigmentation, (iii) staining the animal with ARS, and (iv) clearing the animal of excess stain (244). The ARS molecule is a dihydroxyanthraquinone, likely binding the Ca^2+^ on the hydroxyapatite surface to form either a salt or a chelate form (245), thus it specifically stains mineralized tissue. In disease models ARS will stain ectopic mineralization in soft tissues. For example, ectopic mineralization was shown surrounding the eye, in the wall of the bulbus arteriosus of the heart and in the ventral skin of the dragon fish (*dgf*^−/−^), a knock-out zebrafish model for the gene that encodes Enpp1, and modeled for generalized arterial calcification of infancy (GACI) and pseudoxanthoma elasticum (PXE) (105, 106). Bone collagen in teleosts can also be deposited without being mineralized, as was shown in salmon vertebral bone (246, 247) and in the dentine of replacement teeth of the African bichir (248). It is important to underline that the unmineralized collagen cannot be visualized with ARS, however, mineralization usually quickly follows collagen deposition. Finally, there is also one mineralized collagenous tissue that does not stain with alizarin red S, the hypermineralized enameloid of the tooth cusps (248, 249).

ARS staining for mineralized tissues is frequently used to assess the development of skeletal elements in the head, axial skeleton, and fins at early life stages (Figure 5). In addition, investigating the early skeletal phenotype can be focused on a delay or advance in the development or specifically on the mineralization status of early skeletal elements. Because ARS is autofluorescent in the rhodamine channel (red), it can be used in combination with skeletal transgenic zebrafish reporter lines in which the fluorescent signal of the skeletal cells is in a different light spectrum. Alternatively, a Kaede reporter line, where the spectrum of the fluorescent protein can be changed by exposing the specimen to UV-light, can be used in a more flexible way (196). While most studies using ARS for mineralized tissue examined fixed specimens, ARS can also be used as a live stain especially in early stages where pigmentation does not obscure the underlying skeleton yet [reviewed in (250)]. Staining with ARS can also be employed to assess the juvenile and adult skeleton (Figure 5) because mineralized bone is the main skeletal tissue present at these life stages and is easy to observe with this technique.

#### Alcian Blue

Staining cartilage whole mounts with AB 8GX, similar to ARS staining, is based on several basic steps including (i) removing the pigmentation of the tissue with a bleaching solution (basic pH), (ii) staining the specimens with AB (acid pH), (iii) rehydration and clearing the specimens of excess stain, and (iv) dehydration and storing the specimens. The AB molecule is part of the phthalocyanine dyes with most often copper (Cu^2+^) as the central metallic ion which results in a blue stain. AB has specifically four tetramethylisothiouronium solubility groups with S=C bonds that are easily broken to bind an insoluble AB molecule to the tissue (251). The stain binds as a salt to sulfated and carboxylated acid mucopolysaccharides and glycoproteins present in the cartilage matrix (251). Alcian blue is in most cases dissolved in a dehydrating ethanol/acetic acid solution and brought to a specific low pH. This low pH (1.5–2.5) causes AB to stain very specifically to the cartilage matrix (Figure 5).

Cartilage is the main skeletal tissue in early life stages of zebrafish, particularly in the skull (chondrocranium) and fins (252). Therefore, AB staining has been largely used in early life stages, i.e., 2–20 dpf, to study the morphology of the chondrocranium in different skeletal zebrafish models (62, 68) (Figure 5). Developing malformations are mainly defined as the irregular shape of skeletal elements, but can also be defined by the absence of skeletal elements or the incorrect morphogenesis of a single skeletal element (66, 84). Relative to the entire skeleton, not much cartilage is present in later life stages (late juveniles, adults) of zebrafish, yet AB staining can be used to assess for example cartilaginous joints (92).

#### Alcian Blue/Alizarin Red S Double Stain

Staining of cartilage and mineralized tissues can also be combined in a single specimen, as described in several papers by Kimmel et al. (253, 254). In this protocol tissues are stained first with AB followed by ARS staining (Figure 5). The dual staining for cartilage and mineralized tissues is similar to the single stain methods, except that AB can also be dissolved in a salt/ethanol solution, where the salts can be sodium acetate or the more commonly used magnesium chloride (244, 255).

The dual staining protocol is mostly used to assess development of malformations of the early skeleton but can also be used to investigate the normal development and developmental sequence of the skeleton (69). More specifically, dual staining has been used to assess ossification and mineralization status of cartilaginous bones (40, 87) and shape morphology of skeletal elements (61, 166).

The main advantage of this staining technique is the visualization of both cartilage and bone in an individual specimen, so that both connective tissues can be studied at the same time. However, this approach has also several disadvantages. First, when an acid/ethanol solution is used for AB staining, this acidic staining solution demineralizes the tissues that are subsequently visualized with ARS. This results in a reduced staining of mineralized tissues compromising the correct phenotypic assessment. This issue was reviewed by Witten et al. (24). Therefore, it is advisable to always use single staining protocols, either as an alternative or as a validation method in parallel to the double staining protocol. Second, dissolving AB in a non-acidic salt/ethanol solution is however challenging because pH higher then 6 decreases the specificity of the staining solution for mucopolysaccharides and glycoproteins (251).

#### ARS and AB Whole Mount Staining Advantages and Pitfalls

Considering the simplicity and above all the extensive use of the ARS and AB whole mount staining, a brief overview of its general advantages and disadvantages may be useful.

Both the single staining and double staining approaches are cheap and generally fast to use. Specimens that have not developed scales yet, can often be stained in a single day, with observations made the same day or the day after. In contrast, adult specimens can take up to 2 weeks to stain (244). Indeed, staining protocols need to be adapted to the size of the specimens. Therefore, a thorough description of the staining protocol is indispensable for the interpretation and reproducibility of results (251, 256).

Detailed observations of cartilaginous and mineralized connective tissues can be made owing to the high sensitivity and specificity of both the ARS and AB stains. In particular, small mineralized structures such as the initial mineralizations in early life stages and small intermuscular bones or tendons in adult life stages can be visualized by ARS with high fidelity (24, 234), especially when using fluorescent light which greatly enhances the visibility of these small structures (55, 250). Importantly, ARS stain disappears over time especially in small mineralized structures requiring immediate observation and imaging once the staining procedure is finished. In contrast, when specimens are stored correctly in 100% glycerol, AB staining will remain specific for a longer time (256).

Although AB stains cartilage matrix specifically when the correct pH is used, AB solutions with a pH that is too high or solutions that have a too high or too low salt concentration can result in non-specific staining of non-cartilaginous connective tissue, i.e., collagen type I bone matrix. Non-specific staining can lead to incorrect interpretations of results. Finally, careful interpretation is needed of single AB stained connective tissues in specimens of 15 dpf and older. During the perichondral ossification of cartilaginous bones in zebrafish (Figure 2Bii), when a collagenous sheath forms around cartilaginous bone, the AB solution fails to stain the cartilage, and therefore the cartilaginous connective tissue appears absent. The presence of cartilage beneath the collagen can however still be confirmed using oblique light settings.

### Histological Stains

Bone histology is often necessary to complement other imaging techniques, such as whole mount imaging, and remains one of the methods of choice to investigate the skeletal phenotype and bone mineralization during developmental stages (Table 3). The small size of zebrafish has forced researchers to adapt existing, standard histological procedures performed on human and murine skeletal tissues. High quality histological preparations and extensive knowledge about the zebrafish skeletal anatomy and development are indispensable for a correct skeletal evaluation (36, 45). Since zebrafish share similar bone cell types and cellular markers with mammals, it is possible to apply the standard histological and histomorphometric staining protocols available for mammalian bone, although with some technical optimization. In zebrafish in particular, the cellular composition analysis requires high-magnification imaging because skeletal elements may consist of a very limited number of cells, that are smaller in comparison with mammalian cells (24).

Unlike humans and mice, histology on zebrafish can easily be performed on a whole specimen in different developmental stages. Skeletal development can be followed in early juvenile stages looking at the mineralization of the notochord sheath and of cranial bones, while in adult zebrafish histology is most often performed on the abdominal vertebra (the first 10 vertebrae articulated with ribs, although this number is variable), the scales and the caudal fin rays.

#### Histological Specimen Preparation

In general, the histological procedure for both whole adult zebrafish and dissected bone samples, involves fixation in 4% paraformaldehyde in phosphate buffer saline (PBS) pH 7.2 overnight at 4°C, decalcification in 10% EDTA pH 7.2 for 7 days at 4°C and dehydration according to standard histological protocols or in a gradient series of acetone solutions (199). Importantly, while no decalcification is required up to 20 dpf, for juvenile to adult life stages the time of decalcification varies and depends on the developmental stage and size. Juveniles from 21 dpf till adulthood are normally decalcified for 4 up to 7 days (257).

According to Oralova et al. (199), paraffin embedding does not provide high quality histological details of zebrafish embryos and of early juvenile stages. In these cases, epoxy, or methacrylate resin embedding media are recommended (258). From epoxy blocks, semi, and ultrathin sections can be obtained for light and transmission electron microscopy, respectively, while methacrylate is more suitable for histochemical reactions (24). When using transgenic zebrafish lines expressing fluorescent reporters, fluorescence is generally lost in paraffin embedded samples. Cryosections preserve fluorescence, but significantly decreases the quality of the morphological structure due to processing artifacts. For this reason, Orolova and colleagues developed a new protocol using glycol methacrylate (GMA) embedding, which preserves both fluorescent labeling, epitopes for immunostaining and morphology, making it a more suitable choice (199).

#### Staining of Skeletal Sections

Different stains can be applied to histological sections of the zebrafish skeleton. Masson's trichrome and toluidine blue are commonly used and generally allow visualization of collagen and particular aspects of bone. Masson's trichrome, which usually stains muscle fibers red, collagen and bone in blue/green, cytoplasm in light red/pink, and cell nuclei in dark brown to black, reveals much thinner layers of collagen fibrils in a mutant zebrafish model for type I collagenopathies, a heterogenous group of connective tissue disorders caused by genetic defects in type I collagen (120). Toluidine blue is often used to detect bone cells, but is also a powerful dye to visualize proteoglycans, elastin and, when using birefringent light—collagen type I and type II fiber organization. Toluidine blue was used to detect abnormalities in glycosaminoglycan pattern in the pharyngeal skeleton of a zebrafish model for a recessive OI knock-out of *sec24C/sec24D*, two components of the COPII vesicle complex required for collagen secretion (259). Moreover, sections stained with toluidine blue showed compressed and deformed vertebrae, and excessive bone formation and remodeling at the vertebral endplates in the Bruck syndrome *plod2* mutant, characterized by the loss of type I collagen telopeptide lysyl hydroxylation (16).

The most widely used mineral staining assays include ARS, calcein and von Kossa staining, which specifically bind to calcium in the mineralized bone. In a study by Pasqualetti et al. (260), successive staining with ARS and calcein allowed evaluation of bone formation at the level of the circuli of growing scales in wild-type animals (260). In the *panther* fish, characterized by impaired osteoclast proliferation and differentiation, von Kossa staining enabled detection of altered mineralization of the neural arches (97).

Finally, collagen fiber maturation can be investigated by sirius red staining under polarized light, as performed to study the actinotrichia and lepidotrichia pattern in the *chihuahua* zebrafish, carrying a mutation in collagen type I α1 chain (15, 93, 261).

#### Transmission Electron Microscopy Analysis

Transmission electron microscopy (TEM) has also been used to investigate zebrafish bone. TEM represents a powerful method to analyze ultrastructural features of tissues since it provides much higher magnification and resolution compared to light microscopy, allowing visualization of cellular and matrix structures at a subnanometer scale. For instance, an altered distribution of bone collagen fiber diameter, a frequently described feature in various skeletal pathological conditions, was detected in the *crtap* and *p3h1* knock-out models of OI type VII and VIII by TEM, revealing the crucial role of the collagen post translational modification complex in bone organization (17). TEM was also used to show enlarged endoplasmic reticulum cisterna in these models, reinforcing ER stress as a key element in the OI phenotype and a potential target for new therapeutic approaches (17, 226, 227).

#### Immunohistochemistry

Immunohistochemistry (IHC) on zebrafish sections is also possible but limited, compared to mammal specimens, given the reduced availability of specific zebrafish antibodies. Nonetheless, with IHC, the spatiotemporal pattern of distribution of several proteins, a key prerequisite for understanding development, have been elucidated in embryos both in physiological and pathological conditions (199). For example, a structural defect in the extracellular matrix (ECM) has been detected in the *fndc3a*^wue1/wue1^ zebrafish where IHC of type II collagen showed a loss of mature actinotrichia in 52 h post fertilization (hpf) embryos and β-catenin staining revealed divergent ECM assembly in the regenerated adult fin (262).

Determining the exact spatial localization of the protein of interest in immunostained whole mount larvae is difficult, especially for more deeply located tissues. To overcome this limitation, it is possible to perform whole-mount IHC followed by GMA embedding and sectioning, as was shown by Oralova et al. (199). In this way, the distribution of labeled cells was mapped and quantified allowing for close investigation of the cellular behavior during tissue development, cell migration, and adhesion events, as well as growth and differentiation. As an example, the use of a pan cytokeratin antibody on Tg(*sox17:egfp*) embryos allowed the authors to localize the protein of interest, Sox17, and the epidermis in the same section (199).

Finally, alkaline phosphatase (Alp), expressed by osteoblasts and required for the mineralization of extracellular matrix, and Trap, expressed by osteoclasts, and important for bone resorption, can both be immunostained to detect active osteoblasts and osteoclasts, respectively, and have been used for example to follow cell differentiation in scales (260).

### Histological Analysis of Tissue Regeneration

Zebrafish's ability to repair caudal fin rays and scales has led to the optimization of specific histological protocols for these tissues involving both tissue sectioning as well as whole organ analysis (263). The analysis of histological sections has made clear that during regeneration in the caudal fin rays, cells near the site of injury can dedifferentiate, proliferate and replace the damaged or missing cells (196, 264). Furthermore, histological studies have identified a population of *Runx2/Sp7* positive chondrocytes involved in bone repair, and have helped to elucidate the ability of periosteal cells to generate cartilage in response to injury in *indian hedgehog homolog a* (*Ihha*) mutants (265).

To study mineralization and cellular compositions of caudal fin rays and scales, the tissue can also be isolated and directly stained without the need for dehydration and sectioning. For instance, by using ARS and calcein double staining and ALP immunohistochemistry, the specific mineralization pattern of bone forming cells in different areas of a scale was elucidated (260). Masson's trichrome staining of regenerating ray collagen proved that multiple amputations do not affect the regenerative bone capacity (266).

## Is the Medaka an Alternative Tool in Skeletal Research?

Together with zebrafish, medaka (*Oryzias latipes*) is the other most frequently used small teleost in biomedical research. This species native to East Asia, belongs to the Adrianichthyidae family (order Beloniformes) and had an ancestor living in saltwater (267).

Evolutionarily, zebrafish and medaka are distantly related (268), with the last common ancestor dating back 110–200 million years ago (269). Being a small fish, medaka shares all the advantages already described for zebrafish, although it has a faster generation time, 2 vs. 3 months, shortening genetic experiments (23).

Similar to zebrafish, the medaka shares common skeletal developmental schemes as well as the presence of most of skeletal cells, chondrocytes, osteoblasts, and osteoclasts with tetrapods, but notably is missing osteocytes (23, 24).

The medaka genome, that underwent a whole duplication like that of the zebrafish, is available and easy to manipulate using the same techniques as in zebrafish research allowing easy generation of skeletal disease models and transgenic lines (46, 208, 270–273).

The almost completely conserved phenotypic features between zebrafish and medaka allow researchers to exploit the same imaging techniques to analyze skeletal components in both physiological and pathological conditions, either in terms of x-ray imaging or more specialized methods, such as microCT, whole mount or histological staining methods (23).

## Limitations of the Zebrafish Model

To take full advantage of the zebrafish as a model of human diseases it is important to be aware of existing drawbacks. Due to the extra whole genome duplication compared to mammals, as mentioned above, about 20% of the zebrafish genes have two functional copies, complicating the generation of knock-out disease models (274). Furthermore, some of the duplicated genes have functionally diverged, thus limiting the use of zebrafish in accurately modeling human diseases (11, 24). Additionally, the limited availability of antibodies against zebrafish proteins and the difficulty in establishing tissue specific primary cell lines impairs zebrafish use in research. Finally, the generation of conditional knock-outs and knock-ins is still difficult in zebrafish. Although recently a method to integrate *loxP* sequences at specific sites in the zebrafish genome using the CRISPR/Cas9 technology has been developed, and conditional mutants of *tbx20* and *fleer* have been generated employing Cre recombinase technology (275, 276).

## Conclusions

In the last decade the zebrafish has emerged as a unique model to investigate common and rare human skeletal disorders. The advances in gene editing techniques, from the initial insertion of random genomic mutations by exposure to mutagenic substances, to the knockdown expression of specific genes by antisense morpholino oligonucleotides, to the change of the genome at a specific site by nuclease technologies and their simple use in zebrafish, have all allowed research groups to generate new bone disease models. In particular, the versatile and cheap CRISPR/Cas9 system has found a wide use in many laboratories and undergone a series of optimizations allowing an increasingly specific and error-free gene editing. Nevertheless, its use for knock-in mutations still requires further optimization. The combining of zebrafish skeletal disease models with already available or newly generated transgenic lines, has contributed tremendously to the advances made in *in vivo* analysis of bone cells. The advances in confocal microscopy and the emergence of light sheet microcopy allows for better visualization and characterization of larval phenotypes in skeletal disease models, taking advantage of larvae transparency. X-ray and microCT have been optimized for small adult zebrafish bones, allowing analysis of the whole skeleton or small elements at high resolution. On the other hand, traditional skeletal specific dyes, such as alizarin red and alcian blue remain a valuable tool to study bone in larvae and adults. Finally, biomedical research has an urgent need for high throughput drug screening platforms and zebrafish models of skeletal diseases represent a bridge from *in vitro* to *in vivo* approaches.

In conclusion, ongoing technological advances in analytical techniques are making the zebrafish emerge as a unique and powerful model for the investigation and understanding of human skeletal disorders, and additionally as an efficient platform for compound discovery.

## Author Contributions

FT, JB, RB, AD, AW, and AF: writing—original draft. All authors: review and editing. LL, JB, and AD: figures.

## Conflict of Interest

PS is a Bruker employee. The remaining authors declare that the research was conducted in the absence of any commercial or financial relationships that could be construed as a potential conflict of interest.
